# Remarks upon the Hemostatic Virtues of the Brocchieri Water

**Published:** 1846-08

**Authors:** 


*Remarks upon the Hemostatic Virtues of the Brocchieri Water.*—This celebrated *quack nostrum* was brought to the notice of the Medico-Chirurgical Society of Louisiana, at its sitting in March, by a communication from one of its venders, accompanied by some bottles of the article, with the request that the society would examine and report upon its styptic powers. The society decided (we think very properly) that it would be setting a *bad precedent*, and respectfully declined the offer. Several of the members, however, determined to avail themselves of the earliest opportunity to test the virtues of the remedy, as it had attracted so much notice both in the medical journals and newspapers of the day. We have been kindly favored by Dr. A. Mercier, with the following account of his first experiments with the article, which we have translated from the French:

"*Messrs. Editors.*

"Gentlemen:—At a time when experiments with the '*Eau de Brocchieri*' seem to be the order of the day, permit me to communicate one or two facts which a happy chance threw in my way, and which seem to be of sufficient importance to demand a passing notice.

"Without alluding to all that has been said in relation to the styptic virtues of this water, I resolved to test it for myself; a few days since I was enabled, by good luck, to put the matter to the test of experiment. A French *cuisinier*, when washing a glass, broke it in his hands, and wounded the right index finger. In spite of all the means that were employed on the spot, such as cold water, salt and water, cob-web, the hæmorrhage continued for four hours. At the end of this time he came to claim my assistance, and on removing the dressings, I saw, upon the internal edge of the right index finger, about the middle of the first phalanx, a wound between five and six lines in length, (a piece of the integuments being completely detached) and penetrating beneath the cellulo-adipose tissue which constitutes the pulp of the finger. One of the small anastomotic branches of the index finger was opened, and poured out a constant stream of arterial blood. This I deemed a favourable case for the '*Eau de Brocchieri.*'

"I saturated some lint with this water, and applied it to the wound, recommending the patient to use slight compression. I occasionally poured a small quantity of the Brocchieri water over the lint as it lay on the wound. At the end of seventeen minutes I removed the compress of lint, and found the hæmorrhage as profuse as at first. I then applied two small compresses, one along the internal and the other on the external border of the index, confining both with a bandage. I then lightly cauterised the wound with the nitrate of silver. In five minutes I removed the compresses; the hæmorrhage did not reappear; I then covered the wound with cob-web, confining it with a bandage. I saw the patient nineteen hours afterwards, and no hæmorrhage had reappeared.

"Shortly after this experiment was made, a coloured man presented himself at my office, with a deep wound in the thenar eminence, about an inch and a half in length, in the direction of the axis of the thumb. He had, according to his account, lost about two pounds of blood, and the hæmorrhage was then considerable. In the midst of a mass of muscle which formed a hernia through the wound, I saw the blood spouting, per saltum, from three different points. The muscular branches distributed to the thenar eminence had evidently been opened. After carefully cleansing the wound, I saturated some charpie with the *Eau de Brocchieri*, applied it, and covered the whole with a compress, over which I poured, at short intervals, the *Eau de Brocchieri*. At the end of more than half an hour, in the presence of Dr. Beugnot, who, having heard of my good fortune, came to witness these experiments, I removed the dressings, and we found the hæmorrhage as profuse as before the application. Without pushing these experiments any further, we applied one or two sutures, drew the wound accurately together, and arrested, on the instant, the hæmorrhages. The bleeding did not return. The pain which these two patients experienced from the application of the *Eau de Brocchieri*—a pain incomparably greater than that from the application of strong salt and water, or any other styptic solution, together with its utter inefficacy in cases of hæmorrhage, have induced me to abandon any further trials with it, except, perhaps, in cases of hæmorrhage from *mucous* membranes, as from the nose, rectum, &c. &c., which are so common in this country. MERCIER.

*New Orleans, April 9th,* 1846.

*Ibid.*

